# Flourishing as a highly sensitive person: a mixed method study on the role of nature connectedness and chaotic home environment

**DOI:** 10.3389/fpsyg.2025.1480669

**Published:** 2025-04-16

**Authors:** Susan Carroll, Anna O’Brien, Francesca Lionetti, Anna O’Reilly, Annalisa Setti

**Affiliations:** ^1^School of Applied Psychology, University College Cork, Cork, Ireland; ^2^Department of Brain and Behavioural Studies, University of Pavia, Pavia, Italy; ^3^Environmental Research Institute, University College Cork, Cork, Ireland

**Keywords:** sensory processing sensitivity, nature connectedness, psychological flourishing, home environment, chaos, positive psychology, ageing

## Abstract

**Introduction:**

Theories of Environmental Sensitivity postulate that those who are highly sensitive thrive in favourable contexts and are more disadvantaged by unfavourable ones; however, negative outcomes, instead of positive, are more often investigated. In this study, instead, we focus on human flourishing and what promotes it or hinders it. Recent literature shows that those who are highly sensitive are more connected with nature, and nature connectedness is known to confer psychological benefits. On the contrary, a chaotic home environment is associated with decreased well-being. We hypothesise that a chaotic home environment will negatively impact flourishing, particularly for those who are highly sensitive, while being connected with nature will have a more positive effect on them. Middle and older adulthood are less investigated stages of life.

**Methods:**

856 participants aged 40+ were surveyed on their level of sensory processing sensitivity (SPS), flourishing, current chaos in the home, and nature connectedness. A subsample of 12 highly sensitive people were then interviewed to better understand the role of these dimensions in flourishing as a highly sensitive person.

**Results:**

The results from the quantitative analysis revealed that flourishing was positively associated with nature connectedness and age and negatively with higher SPS. Nature connectedness significantly moderated the relationship between sensitivity /and flourishing, while the level of chaos did not. This interaction was not significant in the youngest (40–49 years) cohort. Qualitative data from interviews with 12 participants provided deeper insights into the challenges faced by highly sensitive individuals, including emotional reactivity and feelings of being different, exacerbated by stressors such as noise and conflict at home. Participants reported significant benefits from nature.

**Discussion:**

Overall, the results showed that connecting with nature significantly contributes to flourishing in highly sensitive individuals, particularly in middle to older age. The findings support the potential of future nature-based interventions to promote flourishing in highly sensitive people.

## 1 Introduction

### 1.1 Sensory processing sensitivity and flourishing

Research has shown that there are individuals with high levels of sensitivity, and this may impact their emotionality and well-being (Lionetti et al., 2024). Understanding the factors that influence their well-being is essential, given the proven heightened risk of mental health issues (e.g., Harrold et al., 2024). This study explores two potential influences—chaotic home environments and nature connectedness—to examine how these factors may hinder or support flourishing in individuals with different levels of environmental sensitivity. The term highly sensitive person (HSP) was first used by Aron and Aron (1997) to describe a temperament trait found in 20–30% of the population whereby individuals display a higher level of sensitivity and responsivity to their environment and adopt a ‘pause to check’ before acting attitude. This trait is studied within the framework of Environmental Sensitivity, as a person’s sensitivity is determined by their difference in reactions to environmental stimuli (Pluess, 2015). The HSP trait is defined as sensory processing sensitivity (SPS) to account for the fact that sensitivity to the environment comprises different levels and should be considered a continuum (Lionetti et al., 2018) measured by the Highly Sensitive Person Scale (Greven et al., 2019). We therefore refer to HSP when we discuss the scores on the scale and SPS when we refer to the trait. Those high in SPS are characterised by deeper cognitive processing of stimuli, emotional reactivity, greater awareness of environmental subtleties, and aesthetic sensitivity (Acevedo et al., 2014; Aron et al., 2012). The trait can confer either advantages or disadvantages depending on an individual’s social and physical environmental circumstances (Pluess, 2015; see Cadogan et al., 2022 for a review). In the extant literature, many studies have focussed on the association between SPS and reduced psychological well-being. Research has found that SPS is associated with reduced life satisfaction in adulthood (Booth et al., 2015), lower subjective well-being (Lionetti et al., 2024; Sobocko and Zelenski, 2015), higher levels of anxiety (Meredith et al., 2016), and depression (Bakker and Moulding, 2012). SPS was also associated with lower quality of life (Costa-Lopez et al., 2021) and stress (Harrold et al., 2024) in two recent systematic reviews. However, in line with the vantage sensitivity (Pluess, 2017) framework, it has been found that in supportive nurturing environments, SPS is associated with higher well-being and highly sensitive people may do better than others in favourable circumstances (Bakermans-Kranenburg and Van Ijzendoorn, 2011; Belsky and Pluess, 2009). Indeed, vantage sensitivity is a theoretical framework proposing that some individuals, particularly those with high environmental sensitivity, are more responsive to positive experiences and interventions and not only to adverse environments (Pluess, 2015; Belsky and Pluess, 2009; Pluess, 2017). This is in contrast with models that focus solely on the negative outcomes of sensitivity, such as diathesis-stress models (Monroe and Simons, 1991) or vulnerability models (Zuckerman, 2004). Empirical studies confirm that high SPS individuals are more responsive to supportive environments. Bakermans-Kranenburg and Van Ijzendoorn (2011) found that highly sensitive children benefited more from supportive environments, and another study (Nocentini et al., 2019) found that highly sensitive children responded more positively to an anti-bullying intervention.

To date, much of the research on high sensitivity looking at positive outcomes has been conducted with children or younger adults, especially students (Booth et al., 2015; Keers and Pluess, 2017), while adults’ research focussed on the negative psychological outcomes (e.g., Harrold et al., 2024). To the best of our knowledge, no study has focussed specifically on middle-aged and older adults exploring determinants of positive outcomes, such as well-being.

Diener (1984) outlined the concept of subjective well-being as a multifaceted construct comprising life satisfaction, positive affect, and low negative affect. The idea of flourishing expands on these basic components by emphasising aspects of well-being that reflect personal growth, fulfilment, and optimal functioning. Flourishing is often measured using validated tools such as the flourishing scale (Diener et al., 2010; Disabato et al., 2019). Flourishing, according to Diener’s scale of flourishing, is a measure of self-perceived success in areas such as relationships, self-esteem, purpose, and optimism, summarised into a single psychological well-being score (Diener et al., 2010). Based on the current literature, one would expect that those who are highly sensitive can flourish when the context is favourable. Although it has been found that the SPS trait tempers with ageing (Ueno et al., 2019), focussing on supporting flourishing across the lifespan highlights the need to investigate SPS in these less explored age groups.

### 1.2 Ageing and sensory processing sensitivity

Middle to older adulthood is an important yet often overlooked stage in research on sensitivity and well-being. First, existing literature on sensory processing sensitivity (SPS) tends to focus on children, university students, or a broad age range, leaving a gap in understanding how sensitivity interacts with well-being across later life stages. Middle adulthood, in particular, is recognised as a period of transition and challenge, often associated with increased stress due to work, caregiving responsibilities, and shifting social roles, which can influence flourishing and mental well-being (Lachman, 2015). Older adulthood brings sensory changes, different opportunities for interaction, and, potentially, physical changes that can affect mobility and independence; therefore, it is important to understand the interplay between ageing and SPS. The present study is a step in this direction.

Ageing presents with challenges, such as decline in social connectedness (Cornwell et al., 2008). However, it can also present opportunities, for example, ageing can be related to increased socialising with neighbours, and partaking in volunteering (Cornwell et al., 2008). Older adults tend to report higher well-being than middle-aged adults (Fields et al., 2022); however, they are at risk of depression (Cai et al., 2023). Poor psychological well-being in older adults may be concurrent with cognitive and sensory impairment that occurs as part of the ageing process; in turn, cognitive and sensory impairment have been shown to increase vulnerability to mental illness (Marin et al., 2011). This indicates that the vulnerability of highly sensitive individuals to the effect of adverse circumstances may increase the risk of being further affected by the challenges of ageing. For example, the risk of developing depression in later life has been positively associated with perceived stress (Cristóbal-Narváez et al., 2022), and higher levels of perceived stress are associated with SPS (Harrold et al., 2024). In addition, when considering negative childhood experiences, higher SPS is associated with having lower life satisfaction later in life (Booth et al., 2015). Furthermore, it is reported that adults with high SPS report poorer physical health compared to their low SPS peers (Kenemore et al., 2023). These risk factors associated with higher SPS can potentially affect flourishing in ageing. However, higher SPS is characterised by higher depth of processing and the ability of building meaningful relationships, which support flourishing in older age (Fastame et al., 2024). Different models (and definitions) of ageing well have been proposed (see Waddell et al., 2025); however, there is scarce evidence of how sensory processing sensitivity interacts with ageing. By focussing on this demographic, this study aims to address this gap by exploring factors potentially impacting flourishing across middle and later adulthood in individuals with different levels of sensitivity.

In summary, a higher level of SPS is frequently associated in the literature with more negative mental health outcomes, due to different aspects of the trait, including the ease of excitation in response to the environment and empathy, potentially leading to compassion fatigue (Fastame et al., 2024). However, it is possible that higher SPS is also associated with positive outcomes, when favourable conditions occur, with reactivity to positive stimuli, depth of processing, and aesthetic sensitivity as potential pathways. Therefore, it is important to determine which favourable or adverse environment surrounding the individual modulates the relationship between the trait and the outcomes (Pérez-Chacón et al., 2021; e.g., Cadogan et al., 2022; Greven et al., 2019 for reviews). In the present study, we focus on two dimensions, one negative, the level of chaos in the home environment, and one positive, the relationship with the natural environment.

### 1.3 Moderating factor: current chaos in the home

Individuals with high sensory sensitivity can be more susceptible to the effects of current chaos in the home (Wachs, 2013) and therefore potentially experience lower flourishing. According to the Conservation of Resources Theory, individuals strive to retain, protect, and build resources that they value, and threat to these resources can lead to stress and decreased well-being (Hobfoll et al., 2018). Research has also emphasised that chronic stress resulting from a chaotic environment (Wachs and Evans, 2010) can exhaust an individual’s coping mechanisms, resulting in detrimental effects on mental health (Edú-Valsania et al., 2022). While some individuals can exhibit resilience in the face of chaos (Edú-Valsania et al., 2022), others experience reduced levels of well-being and psychological distress (Bonanno, 2004). These findings suggest that chaos, by continuously taxing an individual’s resources and coping abilities, can significantly impair their ability to flourish. Given their sensitivity to their environment, it is plausible that highly sensitive individuals would be more negatively impacted by a chaotic home environment.

### 1.4 Moderating factor: connection to nature

Conversely, a potential way to increase well-being may be to capitalise on the aesthetic sensitivity of highly sensitive individuals. Nature connectedness, through the capacity to experience awe (Dunne et al., 2024), could therefore be an avenue to flourishing for highly sensitive people. The salutogenic effects of nature are well known (Setti and Mac Intyre, 2023). Nature has been shown to reduce the effects of stress (Ewert and Chang, 2018). Access to green environments reduces the prevalence of mental health issues in older people (Wu et al., 2015), and spending time in blue and green spaces improves physical, mental, and social health in older adults (Finlay et al., 2015). Setti and Mac Intyre (2023) proposed considering the level of SPS as a new research avenue to maximise nature benefits for well-being. It is important to note that feeling connected with nature predicts well-being independently from the frequency of contact (e.g., Tzankova et al., 2023) and highly sensitive people are more connected with nature (Setti et al., 2022). A series of studies show that higher levels of sensitivity are consistently associated with higher connectedness with nature in different samples (Dunne et al., 2024; Setti et al., 2022), and this connection occurs through multiple pathways (Holzer et al., 2024). Research has also highlighted that nature visits and nature connectedness are negatively related to psychological distress (Mariani Wigley et al., 2025). Therefore, it is plausible that those who are higher in SPS and more connected with nature also report higher levels of flourishing.

### 1.5 The current study

In sum, a chaotic and hyper-stimulating home environment could potentially diminish the level of flourishing, while nature connectedness could be a resource associated with higher flourishing, particularly with increasing levels of sensitivity. These dimensions are particularly important to explore in middle and older age, where life circumstances and the effects of ageing are starting to pose a challenge.

To this end, the study is divided into two parts. The first part examines the relationship between levels of sensitivity, flourishing, chaos in the home environment, and nature connectedness in middle-aged and older adults with a quantitative approach. The second part of the study aims to deepen understanding of how high sensitivity affects well-being in middle-aged and older adults in relation to perceived home chaos and nature connectedness with a qualitative approach.

The following hypotheses were tested:

1. Higher levels of chaos will be negatively associated with levels of flourishing and more so in individuals higher in SPS.
2. Higher levels of nature connectedness will be positively associated with flourishing and more so in individuals higher in SPS.

We will also explore whether these relationships are moderated by age. While no specific hypotheses can be made due to the lack of research on middle and older adults depending on SPS specifically, we advance the tentative hypothesis that highly sensitive individuals will benefit more from nature connectedness, as they have learned what works for them, and potentially, a chaotic home environment could be less impacting with age, for the same reason.

In the qualitative part of the study, as it is aimed at understanding what are the dimensions related to flourishing in the experience of those who are highly sensitive, no specific hypotheses are advanced. However, the interview touches on the moderating factors tested in the quantitative part to contextualise the quantitative data within the qualitative experiences of highly sensitive individuals.

## 2 Method

### 2.1 Design

A mixed methods approach was chosen to benefit from the strengths of both qualitative and quantitative data, allowing for a comprehensive understanding of the research problem (Creswell and Plano Clark, 2017). This approach provides triangulation within the study, where findings from different methods validate each other, and complementarity, where quantitative data can highlight generalisable patterns while qualitative data provide deeper context. A convergent parallel design was used, wherein quantitative data were analysed simultaneously with the qualitative data to cross-validate findings from different perspectives and provide context to the quantitative hypotheses/data through a qualitative exploration (Creswell and Plano Clark, 2017). Quantitative data were gathered first, and participants were then given the opportunity to sign up to partake in a qualitative interview at a later date.

### 2.2 Participants

#### 2.2.1 Quantitative

For part one of this study (quantitative), convenience sampling was used to recruit participants (*N* = 1,092). Participants with less than a 100% response rate on the survey items were excluded from the final sample size of 856 participants, as an initial analysis indicated that missing responses were not random (MCAR *p* < 0.001). Further inspection of the data showed that missing responses were primarily in the HSP scale items 1, 2, 3, 5, 7, and 9. Participants with missing values were therefore excluded from the analysis.

Participants were recruited based on age (aged 40+) through social media platforms and internal staff emails at University College Cork and were asked to complete an online survey that took approximately 10 min.

The age ranges of participants were captured in three categories: 40–49, 50–59, and 60+ (see Table 1 for demographic characteristics). Participants varied in terms of demographics, with a range of ethnicities and nationalities; however, the majority of participants were residing in Ireland or the UK at the time of the study. On completion of the survey, participants were asked to provide email details if they would be willing to participate in an interview on the topics of the survey.

**Table 1:** Descriptive statistics for demographic variables.

	Category	*n*	%
Age	40–49	379	44.3
	50–59	329	38.4
	60+	141	16.5
Sex	Female	716	83.6
	Male	131	15.3
	Non-binary	3	0.4
Level of education	Prefer not to say	36	4.2
	Primary	7	0.8
	Post-primary	234	27.3
	Bachelor’s degree	314	36.7
	Master’s degree or higher	259	30.3

#### 2.2.2 Qualitative

Participants for the second part of the study (*N* = 12) were chosen from those (*N* = 235) who provided their email details, based on the criteria of having the highest mean HSP scores, indicating higher SPS, being aged 50+, and not having prior knowledge of SPS as an innate personality trait. The rationale for this choice was due to further research questions not explored here. These inclusion criteria resulted in a sample size of 12 participants (10 female and 2 male participants). The 12 participants who, when contacted, agreed to be interviewed were scheduled to participate in a semi-structured interview online. The study was approved by the Ethics Committee of the School of Applied Psychology (subcommittee of Social Research Ethics), University College Cork.

### 2.3 Measures

#### 2.3.1 Levels of sensitivity

The Highly Sensitive Person Scale – Brief Version (HSP-12) (Pluess et al., 2023) was utilised as it is a validated and frequently used scale in the literature. This 12-item scale measures participants’ responses with a 7-point Likert scale in which 1 = not at all and 7 = extremely. Higher HSP-12 scores indicate greater sensory sensitivity, which is associated with sensitivity to subtleties in the environment, emotional reactivity, and aesthetic sensitivity, while lower scores are associated with resilience to environmental inputs (see Greven et al., 2019). The scale has a Cronbach’s alpha of *α* = 0.81. Examples of items in HSP-12 include “*do you seem to be aware of subtleties in your environment?*” and “*Do you find it unpleasant to have a lot going on at once?*”

#### 2.3.2 Well-being

The Flourishing Scale (FS) (Diener et al., 2010) was used to measure levels of well-being. This is an 8-item, 7-point scale measuring well-being across eight key indicators: having a sense of purpose and meaning in life, positive relationships, engagement in daily activities, contributing to others’ well-being, competence, self-acceptance, optimism, and feeling respected or having a sense of belonging. It is a score of well-being in which higher scores indicate greater psychological resources and strengths, with items such as ‘*I lead a purposeful and meaningful life*’ and *‘My social relationships are supportive and rewarding*’. The FS had a Cronbach’s alpha of *α* = 0.82 in a sample of older adults (Fassih-Ramandi et al., 2020). The scale has a Cronbach’s alpha of α = 0.88 in our sample.

#### 2.3.3 Chaos in the home environment

The Confusion, Hubbub, and Order Scale (CHAOS) Scale (Matheny et al., 1995) is designed to assess the level of disorganisation and confusion in the home environment (e.g., ‘*we almost always seem to be rushed*’; ‘*you cannot hear yourself think in our home*’). This is a 15-item, true or false scale. Higher scores equal greater chaos (more negative lived environment). Cronbach’s alpha of the CHAOS Scale is α = 0.82.

#### 2.3.4 Connection with nature

Levels of nature connectedness were measured using the Nature Connection Index (NCI) (Richardson et al., 2019). This is a 6-item measure of relationship with nature using a 7-point Likert Scale with 1 *being completely disagree* and 7 *being completely agree*. Each item response on the NCI has different weighting points, which altogether add up to 100%; examples include ‘*I always find beauty in nature*’ (Weight = 15%) and ‘*I feel part of nature*’ (Weight = 23%); scores were adjusted accordingly and added up; the higher the score, the greater the connection with nature. NCI has a Cronbach’s alpha of α =0.91.

#### 2.3.5 Semi-structured interviews

In the second part of the study, in-depth semi-structured interviews were conducted online using the Microsoft Teams platform. Each interview session began with a short explanation of high sensitivity as a temperament trait; this was followed by a set of open questions about the experience of being highly sensitive, conditions in the home environment, and relationship with nature (see Supplementary Material for interview schedule).

### 2.4 Analysis

#### 2.4.1 Part 1: quantitative

In the first part of the study, descriptive statistics explored the mean, median, standard deviation, and total score of the HSP-12, the Flourishing Scale, the CHAOS Scale, and the Nature Connection Index, as well as identifying the age range of the cohort, the levels of education achieved, and gender. A Spearman correlation was used to assess the relationship between the variables used in the study due to the inclusion of categorical variables. Multiple regressions were used to test the hypotheses.

#### 2.4.2 Part 2: qualitative

In the second part of the study, the interview data were explored using the six-phase process of thematic analysis (TA) (Braun and Clarke, 2024). First, the coder (SC) familiarised with the transcripts, derived initial codes, and then identified recurrent patterns, which were collated into themes, ensuring that the voice of all participants was represented. An inductive approach was adopted; however, it was guided by the research questions. The main coder (SC) recognised themselves as a highly sensitive person, which should be acknowledged in thematic analysis (Braun and Clarke, 2019; Clarke and Braun, 2017). The senior author (AS) provided guidance during this process; the themes were then discussed with AOT to further check on the process.

## 3 Results

### 3.1 Quantitative analysis

#### 3.1.1 Descriptive statistics

In a sample of 856 participants, the majority (44.3%) were aged between 40 and 49, followed by 38.4% aged 50–59, and 16.5% aged 60 or older. The sample was predominantly female (83.6%), with 15.3% male and 0.4% identifying as non-binary. In terms of education, 36.7% of participants had a bachelor’s degree, 30.3% had a master’s degree or higher, 27.3% had completed post-primary education, 0.8% had primary education, and 4.2% preferred not to disclose their level of education. See Tables 1, 2 for descriptive statistics.

**Table 2:** Descriptive statistics for scale variables.

	Min	Max	M	SD
HSP-12 scale	12	84	51.64	11.45
Flourishing scale	11	56	46.53	6.43
CHAOS scale	2	13	7.45	1.45
NCI	0	100	71.26	26.15

M = mean; SD = standard deviation. HSP-12 = Highly Sensitive Person Scale-Brief Version; NCI = Nature Connection Index; CHAOS = Confusion, Hubbub, and Order Scale.

#### 3.1.2 Correlation

The data were converted in Z-scores because Z-scores standardise data by converting the original values into units of standard deviations from the mean. This makes the variables calculated on different scales easier to compare. Correlation analysis revealed several significant relationships among the variables, displayed in Table 3. Flourishing was significantly positively correlated with nature connectedness (NCI) (*r* = 0.29, *p* < 0.01) and age (*r* = 0.18, *p* < 0.01). However, there was no significant correlation between Flourishing and CHAOS (r = 0.04). Flourishing also had a significant negative, although small, correlation with the Highly Sensitive Person (HSP) scale (*r* = −0.14, *p* < 0.01). The HSP-12 scale showed a significant positive correlation with the NCI (*r* = 0.20, *p* < 0.01) but no significant correlation with CHAOS (*r* = 0.12, *p* < 0.01). It also had a significant negative correlation with gender (*r* = −0.16, *p* < 0.01), with female as the reference group. In addition, the NCI had a significant positive correlation with age (*r* = 0.11, *p* < 0.01) but no significant correlation with CHAOS (*r* = 0.20, p < 0.01). Finally, CHAOS was significantly positively correlated with education (*r* = 0.08, *p* < 0.05).

**Table 3:** Spearman’s rho correlations.

Measure	1	2	3	4	5	6	7
1. Flourishing	-						
2. HSP-12	−0.14**	-					
3. NCI	0.29**	0.20**	-				
4. CHAOS	0.04	0.12**	0.20**	-			
5. Gender	−0.03	−0.16**	−0.05	0.04	-		
6. Age	0.18**	−0.002	0.11**	0.05	0.05	-	
7. Education	0.04	0.05	−0.08*	0.08*	0.15**	−0.05	-

*Statistical significance is indicated as *p < 0.05, ** *p* < 0.01, and ***p < 0.001; HSP-12 = Highly Sensitive Person Scale-Brief Version; NCI = Nature Connection Index; CHAOS = Confusion, Hubbub, and Order Scale.

#### 3.1.3 Effect of NCI and HSP on flourishing

The initial regression analysis (linear regression, with predictors entered simultaneously), displayed in Table 4, was significant [*F* (8, 839) = 32.24, *p* < 0.001] and showed a significant negative association between gender and flourishing (*β* = −0.10, t = −3.36, *p* < 0.001), indicating that female participants reported higher levels of flourishing. Age had a positive impact on flourishing (β = 0.09, t = 2.96, *p* = 0.003), suggesting that older individuals tend to experience higher flourishing. CHAOS significantly negatively impacted flourishing (*β* = −0.25, t = −7.49, *p* < 0.001), indicating that higher levels of CHAOS are associated with lower flourishing. The interaction between HSP-12 and CHAOS did not reach significance (β = −0.06, t = −1.90, *p* = 0.058). Higher sensitivity (HSP-12) negatively predicted flourishing (*β* = −0.18, t = −5.63, *p* < 0.001). Nature connectedness (NCI) had a significant positive effect on flourishing (β = 0.28, t = 8.64, *p* < 0.001). In addition, the interaction between HSP-12 and NCI was significant, positively predicting flourishing (*β* = 0.11, t = 3.46, *p* < 0.001).

**Table 4:** Regression model of age, gender, education, HSP-12, NCI, CHAOS, and their interactions as predictors of flourishing.

Predictor	B	*SE* B	*b*	*t*	95% CI		*p*
					*LL*	*UL*	
(Constant)	−0.10	0.15		−0.66	−0.38	0.19	0.509
Gender	−0.27	0.08	−0.10	−3.36	−0.43	−0.11	<0.001
Age	0.13	0.04	0.09	2.96	0.04	0.21	0.003
Education	0.06	0.03	0.06	1.91	−0.00	0.12	0.056
HSP-12	−0.18	0.03	−0.18	−5.63	−0.24	−0.12	<0.001
CHAOS	−0.25	0.03	−0.25	−7.49	−0.31	−0.18	<0.001
NCI	0.28	0.03	0.28	8.64	0.21	0.34	<0.001
HSP-12*NCI	0.10	0.03	0.11	3.46	0.05	0.16	<0.001
HSP-12*CHAOS	−0.06	0.03	−0.06	−1.90	−0.13	0.00	0.058

HSP-12 = Highly Sensitive Person Scale-Brief Version; NCI = Nature Connection Index; CHAOS = Confusion, Hubbub, and Order Scale.

#### 3.1.4 Nature connectedness and HSP groups

**Figure 1:**
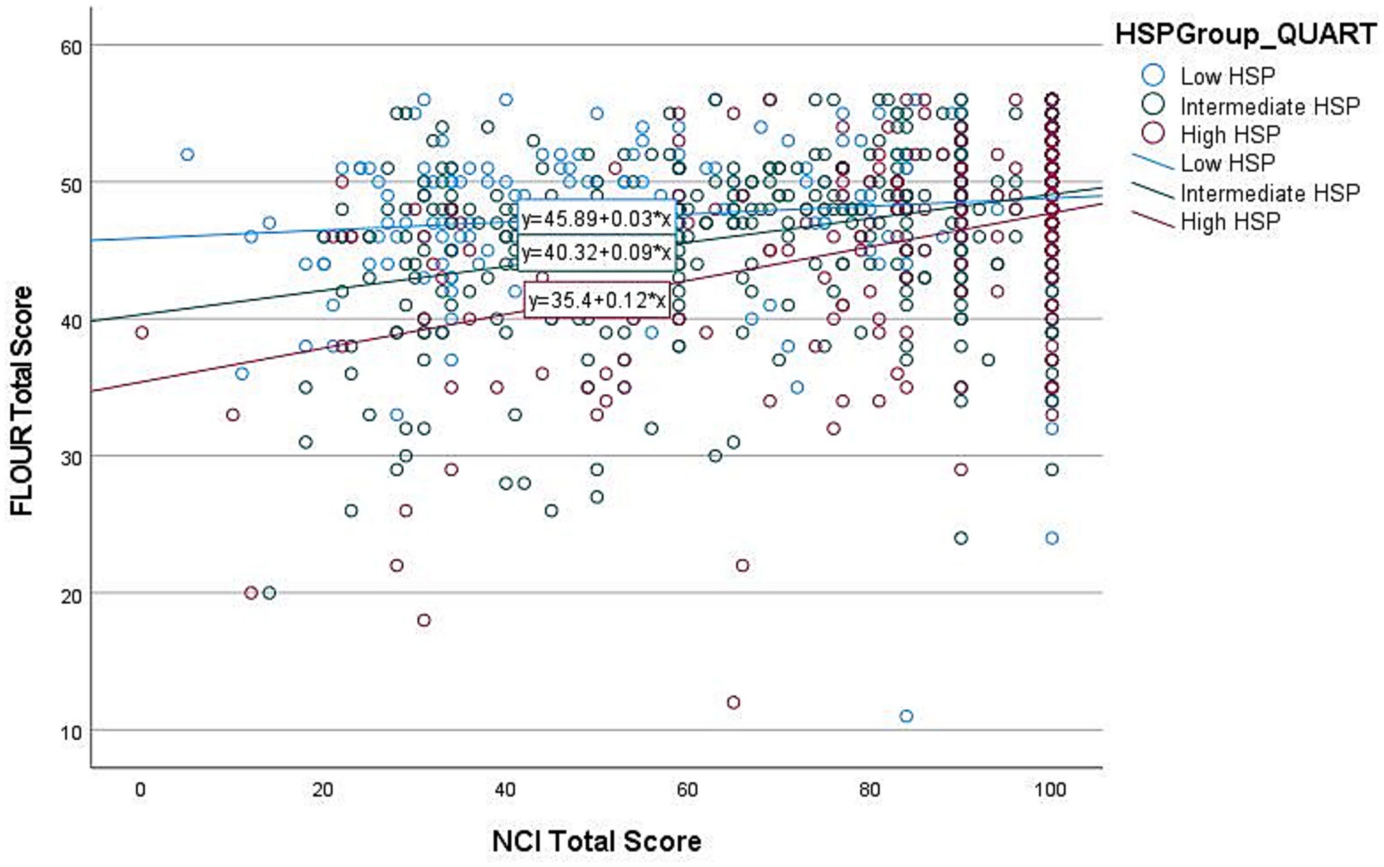
Individual HSP group effects of NCI on Flourishing.

To further explore the HSP-12*NCI interaction, we conducted separate multiple linear regressions (see Table 5) with flourishing as the dependent variable and NCI as the predictor variable, split by low, intermediate, and high HSP groups. HSP-12 was divided into three levels: low (first quartile: HSP-12 score of 0 to 43), intermediate (second and third quartile: HSP-12 score of 44 to 60), and high (fourth quartile: HSP-12 score of 61 to 84) according to Lionetti et al. (2018). All models were significant: low HSP (*F*(1, 210) = 4.117, *p* = 0.044), intermediate (*F*(1, 438) = 66.247, *p* < 0.001), and high HSP groups (*F*(1, 201) = 39.057, *p* < 0.001) (see Figure 1 for the interaction).

**Table 5:** Regression model of NCI as a predictor of flourishing and HSP group (scores on HSP-12).

	Predictor	B	*SE* B	*b*	*t*	95% CI		*p*
						*LL*	*UL*	
Low HSP	(Constant)	0.22	0.06		3.37	0.09	0.34	<0.001
	NCI	0.12	0.06	0.14	2.03	0.00	0.24	0.044
Intermediate HSP	(Constant)	−0.01	0.04		−0.14	−0.09	0.08	0.892
	NCI	0.36	0.04	0.36	8.14	0.27	0.44	<0.001
High HSP	(Constant)	−0.38	0.08		−4.80	−0.53	−0.22	<0.001
	NCI	0.50	0.08	0.40	6.25	0.34	0.66	<0.001

NCI = Nature Connection Index.

#### 3.1.5 Nature connectedness, HSP-12, and age

As we were also interested in understanding the role of age in modulating the relationship between nature connectedness and levels of sensitivity in relation to flourishing, the impact of age was then analysed through a regression model, with an added interaction effect of HSP-12*NCI*Age. The initial model was significant (*F* (4, 843) = 38.776, *p* < 0.001), with the model showing a significant three-way interaction HSP-12*NCI*Age (β = 0.134, *p* < 0.001) (see Table 6).

**Table 6:** Regression model: HSP, NCI, age, and HSP*NCI*Age interaction as a predictor of flourishing.

Predictor	B	*SE* B	*b*	*t*	95% CI		*p*
					*LL*	*UL*	
(Constant)	−0.36	0.08		−4.41	−0.51	−0.20	<0.001
HSP-12	−0.21	0.03	−0.21	−6.39	−0.27	−0.14	<0.001
NCI	0.32	0.03	0.32	9.67	0.25	0.38	<0.001
Age	0.19	0.04	0.14	4.27	0.10	0.27	<0.001
HSP*NCI*Age	0.07	0.02	0.13	4.17	0.04	0.10	<0.001

HSP-12 = Highly Sensitive Person Scale-Brief Version; NCI = Nature Connection Index.

Separate multiple regressions were then conducted on each age group to explore the interaction (see Supplementary Tables 1–3 for details).

##### 3.1.5.1 Age group 40–49

**Figure 2:**
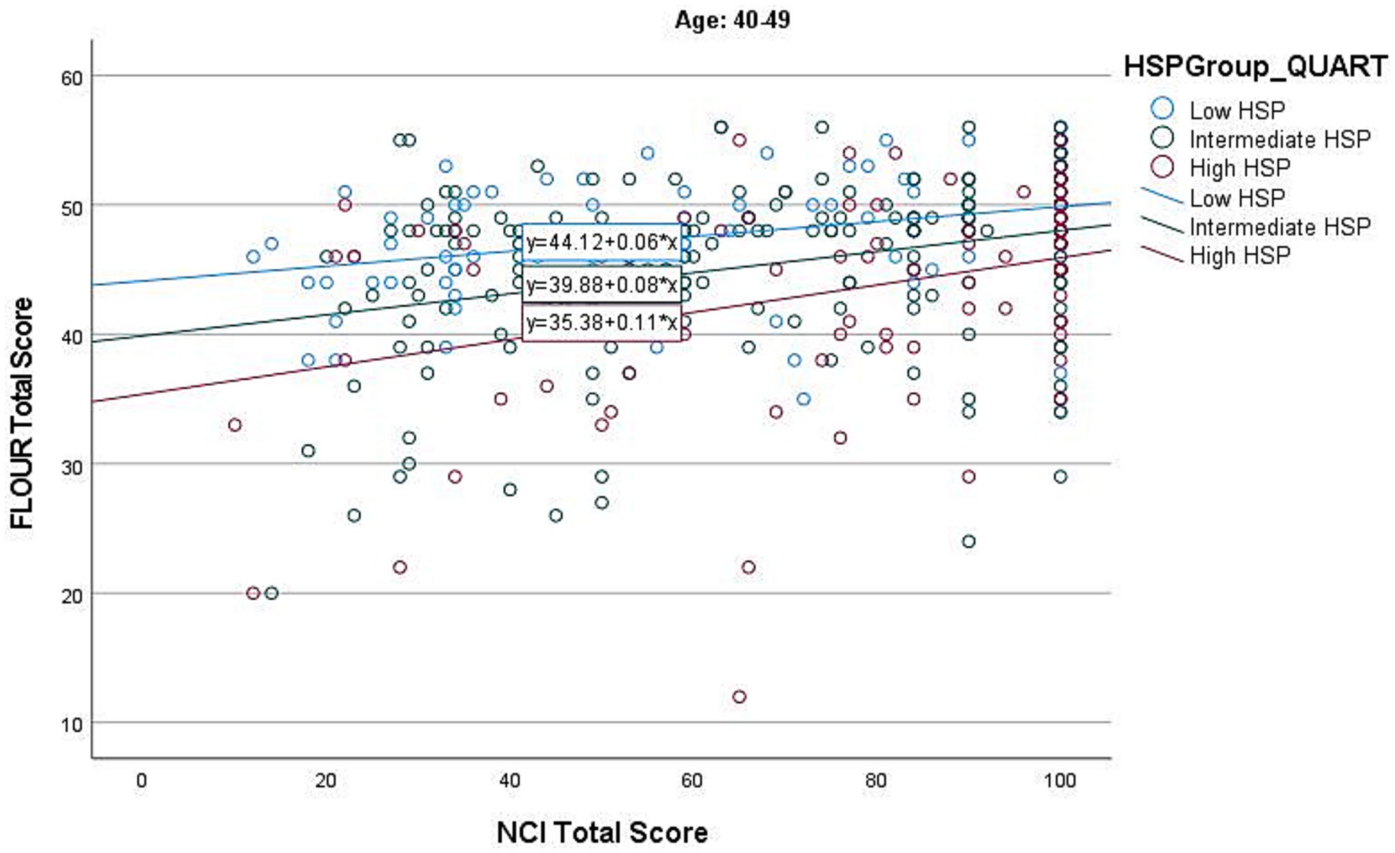
Individual HSP group effects of NCI on Flourishing in age group 40–49.

The regression model for the 40–49 age group was significant (*F*(3, 374) = 22.141, *p* < 0.001). While both the HSP group and NCI had a significant impact as individual variables, the HSP-12*NCI interaction was not significant (β = 0.049, *p* = 0.306) (see Figure 2 and Supplementary Table 1).

##### 3.1.5.2 Age group 50–59

**Figure 3:**
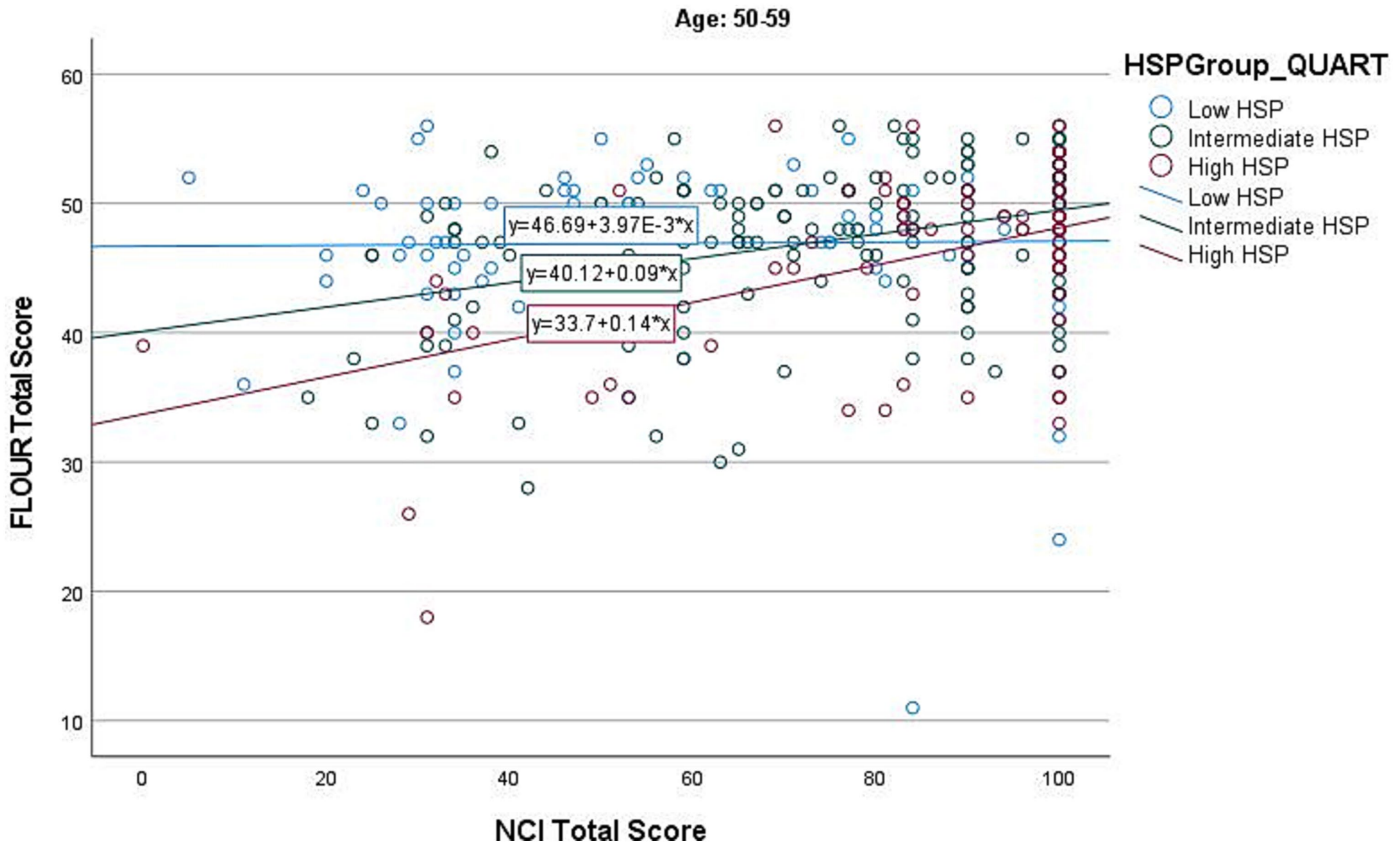
Individual HSP group effects of NCI on Flourishing in age group 50–59.

The regression model for the 50–59 age group was significant (*F*(3, 325) = 15.354, *p* < 0.001). Figure 3 shows a common trend for those in the intermediate and high HSP groups. The interaction between HSP-12 and NCI was significant (β = 0.19, *p* < 0.001) (see Supplementary Table 2).

##### 3.1.5.3 Age group 60+

**Figure 4:**
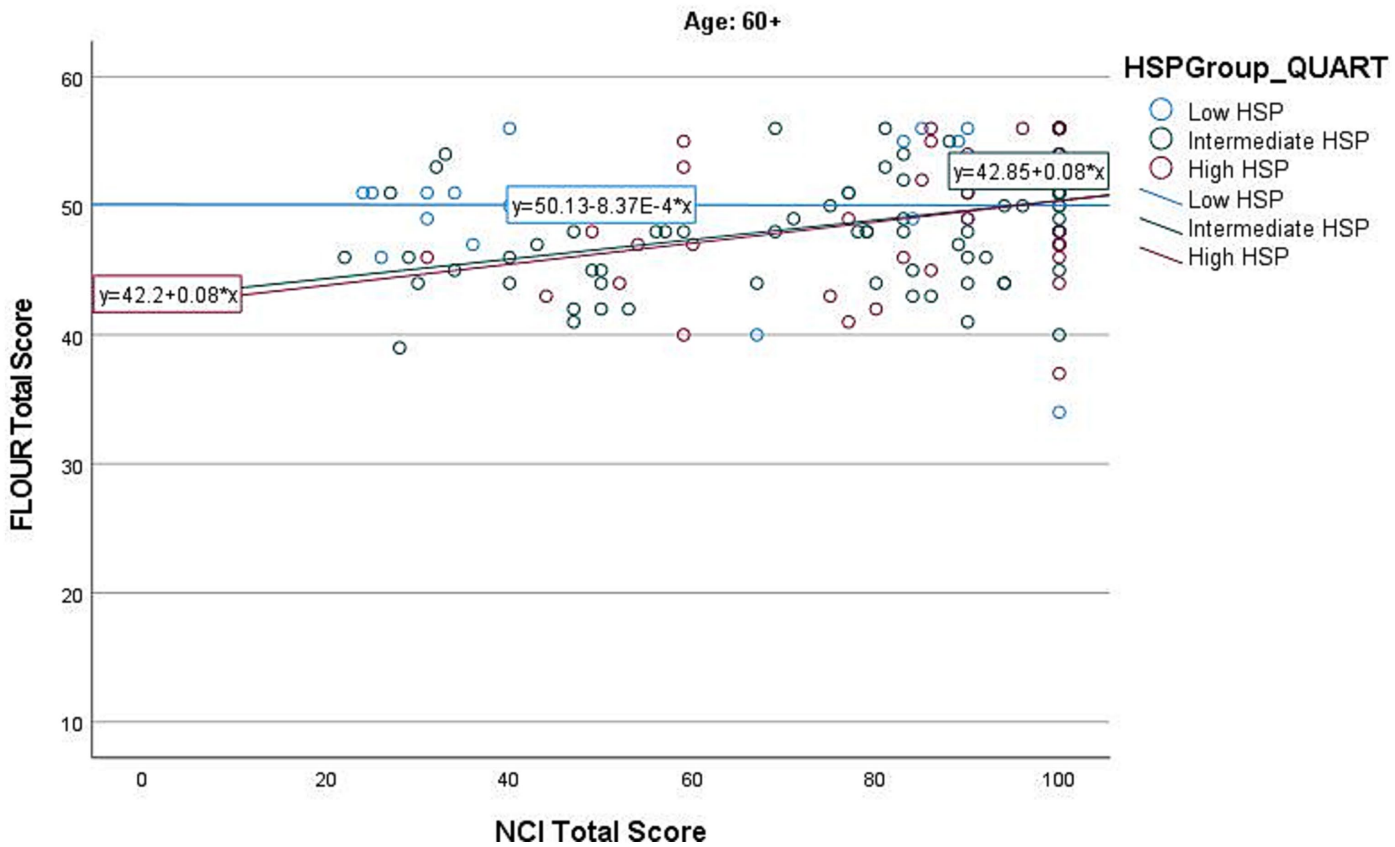
Individual HSP group effects of NCI on Flourishing in age group 60+.

The regression model for the 60+ age group was significant (*F*(3, 137) = 5.925, *p* < 0.001), with a significant interaction between HSP-12 and NCI (β = 0.19, *p* = 0.028) (see Supplementary Table 3 and Figure 4).

### 3.2 Qualitative analysis

**Figure 5:**
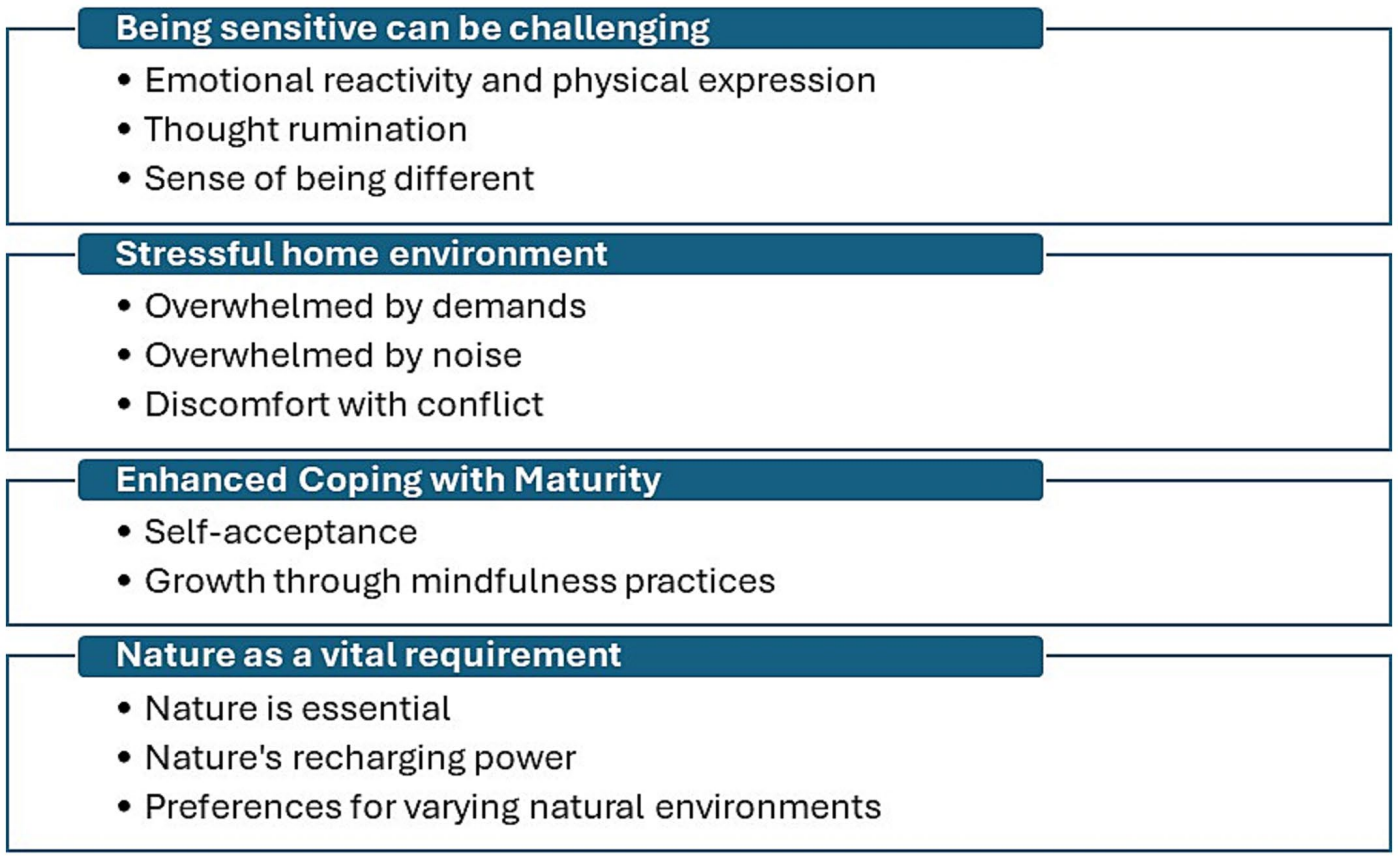
Themes and sub-themes.

Four main themes were identified, with two or three subthemes within each theme (see Figure 5). Themes included the challenges of being highly sensitive and the role of nature in maintaining well-being.

#### 3.2.1 Theme 1: being sensitive can be challenging

All participants (*n* = 12) identified factors related to being sensitive that are challenging for their well-being. Within the main theme, three subthemes were identified.

##### 3.2.1.1 Subtheme 1.1: emotional reactivity and physical expression

The first subtheme illustrates one of the strong emotional reactions that can be seen in sensory processing sensitivity. This strong emotional reaction, becoming upset or crying easily, was recognised by some participants and/or others around them. A uniquely Irish expression to represent this is that of “your bladder is very close to your eyes” (P_4_) which is often attributed to people who become upset and cry easily and was reported by one participant as being attributed to them by family members, while other participants reported, being “*incredibly emotionally sensitive*” or that they could “*cry in an instant*” (P_7_), or reported “*feeling more emotional about things than others*” (P_2_) or “*taking things to heart”* (P_3_).

##### 3.2.1.2 Subtheme 1.2: thought rumination

Another challenge, reported by half of the participants (*n* = 6), was the tendency to ruminate *“would ruminate over things in my head for a long time,”* (P_2_). One participant noted, *“If there was an issue, I took it to bed with me.” “It wrecked me”* (P_4_). One participant remarked, *“I’d brood on things more than other people”* (P_10_), while two other participants mentioned how they *“internalised emotions”* (P_7_) and (P_8_) instead of expressing their feelings, saying, *“I probably did not speak up and say, that’s too much for me.”* (P_7_).

##### 3.2.1.3 Subtheme 1.3: sense of being different

The third subtheme for the majority of participants (*n* = 11) was the feeling of not fitting in with others. Participants described feeling different from those around them, for example, one participant stated that they “*would have described self as sensitive or odd compared to others*” (P_1_), while another participant reported that they “*always had a sense of not fitting in, thought it was just me being weird*” (P_7_). In many instances, this manifested as individuals thinking that there was something wrong with them, “*You do wonder and think, oh, what’s wrong with me”* (P_10_).

#### 3.2.2 Theme 2: stressful home environment

As well as challenges, all 12 participants described some key conditions in the home environment that caused them stress such as too much activity, loud noise, or tension.

##### 3.2.2.1 Subtheme 2.1: overwhelmed by demands

All participants noted becoming very stressed when they had too much activity with little downtime or breaks. This especially resonated with one participant who stated “*I’m not the sort of person who can run on busy, busy, busy all the time, there has to be a quiet day just to backpedal a bit*” (P_11_). For others, there is a general dislike of too much activity, as one participant reported “*I like to have time to stop and gather myself*” (P_10_), or another stated “*I do not like having to rush from one thing to another*_.”_ (P_7_).

Participants described being “*overwhelmed*” (P_2_ and P_3_) when they are very busy and described being overscheduled as “*stressful, 100%”* (P_5_) and reported “*feeling calmest when the schedule is done*.” A method to mitigate overwhelm in her daily routine for one participant was to ensure she was “*very organised”* (P_4_).

##### 3.2.2.2 Subtheme 2.2: overwhelmed by noise

Noise was another influencing factor in the home environment that participants identified as a significant cause of discomfort. Participants described feeling a sensory overload if there was too much volume of noise, or if participants heard multiple sounds at once. “*I cannot cope with too much noise, I cannot cope with two noises together, like if the TV is on and the radio*” (P_3_), “*I could live without a television perfectly happy, yeah, the noise of it would stress me out*” (P_9_), with some participants noting noise as a source of irritation and annoyance “*If someone puts on the radio in the morning, I just do not want to hear it*” and “*get very annoyed and irritated”* (P_7_). Some participants identified certain noises that they were extremely sensitive to. For example, one participant stated, “*My sister says mine is the house where we have to suck crisps”* (P_3_). In addition, medial daily noises are a source of significant annoyance: “*breathing, chewing and paper rustling”* “*I cannot bear it, I cannot bear it”* (P_5_).

##### 3.2.2.3 Subtheme 2.3: discomfort with conflict

Tension was another stressor identified in the home environment by five of the participants. For example, one participant reported, “*When I hear people arguing, it affects my mental balance*” (P_9_), and “*Any arguments, I would find very stressful, I feel as if my nerve endings are all jangly*” (P_11_), whereas for another participant the tension, or conflict, got too much to stay in the environment “*the kids arguing, I was overwhelmed, I just had to go*” (P_3_).

#### 3.2.3 Theme 3: enhanced coping with maturity

All participants reported that they adapted and coped better with the challenges and stresses as they matured and had more life experience.

##### 3.2.3.1 Subtheme 3.1: self-acceptance

Individuals reported being more accepting of themselves as they matured. For example, one participant reported that “*I am happy with my authentic little self now”* (P_1_), whereas another participant noted that “*As I’ve gotten older, I’m better with it, but probably when I was younger, it was harder”* (P_2_). One participant noted that they now prioritise and look after their needs: “*I do not feel obliged anymore to be in environments, I know will be hectic or busy”* (P_3_). Others pointed out, “*I’ve adapted my own little ways”* or “*try to pre-empt things”* (P_6_), while one reported that she now has “*more of an understanding of who I am.”* (P_1_).

##### 3.2.3.2 Subtheme 3.2: growth through mindfulness practices

Knowledge and experience gained through personal development practices were noted to have helped 10 of the 12 participants to cope and adapt. The experience and knowledge gained from meditation formed a crucial part of this practice. For example, one participant noted, *“I found I was burning out, because I did not have the balance right, I meditate*” (P10), and “*The calm and peace the meditative state reveals is very valuable to me*” (P_8_). Meditation as a practice was so important for one participant they reported “*I brought meditation into my daily life, so I would be able to function: It worked”* (P_4_) and “*I’m studying mindfulness*” (P_1_).

#### 3.2.4 Theme 4: nature as a vital requirement

For all participants, nature was a vital requirement for their well-being.

##### 3.2.4.1 Subtheme 4.1: nature is essential

Participants identified nature as being essential to their well-being. Accessing nature was very important, “*It’s a priority”* (P_9_), as one participant stated, “*I would need it, even if it’s just sitting in the garden for 10–15 min”* (P_1_), whereas another stated, “*I need nature definitely, need access to nature close to my home.”* (P_2_). In different ways, participants indicated how vital nature was for them, for example, describing that “*Nature is very important, very important, I’ll emphasise that”* (P_10_), or that “*I feel much better when I am outside*” (P_10_).

##### 3.2.4.2 Subtheme 4.2: nature’s recharging power

Participants reported a number of significant benefits that they gained from connection with nature. In different ways, participants felt replenished; “*It’s like you know you plug in your phone to recharge, I feel depleted if I do not have it”* (P_1_), or it “*makes me feel reset*” (P_2_), “*I love to walk in nature, everything about it soothes my body and my mind*,” (P_9_). It had a therapeutic and/or restorative value for some participants; “*It’s the best therapy, I have*” (P_4_), “*It makes me feel amazing, it restores my equilibrium*” (P_7_) or “*I feel energised, refreshed*” (P_10_) after being in nature.

##### 3.2.4.3 Subtheme 4.3: preferences for varying natural environments

All participants identified certain favourite places within nature. The majority of participants (*n* = 10) chose being by the sea as their favourite place in nature; “*I’ve always been drawn to the sea, it’s that expanse*” (P_2_), or another participant who stated, “*I love everything about the sea, it just hits the right spot for me*,” *“the sea is my area, I just love the sea”* (P_6_), *“my preference would be the beach”* (P_1_). Forests, woodlands, and hills were also identified by seven participants as a favourite place at times, with some saying that it depends on what they need at a particular time, “*If I need to release, I get into water, if I need grounding, it’s the forests and the green”* (P_7_) and the feeling of a particular place, “*It depends on the feeling I get from a place, there are certain woodland areas that tick the box as well*” (P_2_). A number of participants also reported that even small amounts of nature are enough to help them feel better, in times they cannot get out into bigger nature. For example, one participant stated “*Once I got my 10 min out in the little* [courtyard garden in hospice] *area, I was OK*” (P_1_), whereas another participant stated, “*I like just sitting looking out at the garden”* (P_6_) and reported that “*I have lovely pots with lovely colour*” [to look out at] *“it is peaceful and nice”* (P_9_). Even short periods of time or just a quick visit to a garden were restorative here as one participant remarked “*It does not matter if it is just a friend’s garden or even a little pot plant”* (P_10_) or another stated, “*Gardening is my go to thing, I do not need to climb* [a mountain] *or anything like that*” (P_5_).

## 4 Discussion

This mixed methods study addressed flourishing in individuals with different levels of sensitivity, with a specific focus on those who are middle-aged and older. While the literature highlights the disadvantages of being highly sensitive for well-being (Greven et al., 2019; Harrold et al., 2024), we focussed on flourishing and the factors that can promote or hinder it, depending on SPS. We considered two factors, namely, a self-reported chaotic home environment and being connected with nature. We hypothesised that the first may be detrimental for highly sensitive people due to the low sensory thresholds and ease of excitation; while the second can support well-being and enhance the benefits derived from the natural environment, given the aesthetic sensitivity of highly sensitive individuals. In the first part of the study, we tested the hypotheses that a chaotic home environment would moderate negatively the association of sensitivity with flourishing and that nature connectedness would moderate it positively. In the second part of the study, we delved more in-depth into the experience of being highly sensitive in relation to these two factors.

The findings of the quantitative part of the study support the hypothesis that higher levels of nature connectedness are associated with higher flourishing in those with higher levels of sensitivity. While a higher score on the HSP-12 scale is associated with lower flourishing, this is not the case in those who are more connected to nature; therefore, nature connectedness helps highly sensitive people to flourish. This is supported by the qualitative findings of the study, where participants consistently reported seeking nature to improve their well-being, relieve stress, and consider it an essential part of their lives. The mechanism through which nature connectedness enhances flourishing in high SPS individuals is likely multifaceted.

First, nature connectedness is associated with hedonic well-being and life satisfaction (Capaldi et al., 2014), as well as eudemonic well-being (Pritchard et al., 2020). Martin et al. (2020) found that nature connectedness was associated with eudemonic well-being, when controlling for nature contact and socio-demographic factors in a large sample of participants. In the same study, nature connectedness moderated the relationship between some types of exposure and well-being. Along this line, a potential pathway through which nature connectedness enhances flourishing in highly sensitive people is feeling part of nature as a meaningful experience, enhancing eudemonic well-being and providing positive emotions. Participants in the qualitative part reported that nature is a requirement in their lives, suggesting a deep meaning to their relationship with nature. This is reflected in the multifaceted appraisal of the benefits of different kinds of environment. It also aligns with the findings of Dunne et al. (2024) where, in a large sample of participants, higher sensory processing sensitivity was associated with higher nature connectedness.

Second, the positive emotions experienced when in nature could constitute a way to enhance nature connectedness and therefore enhance well-being, through positive memories (e.g., Cadogan et al., 2023), or by bolstering the effects of limited exposures to nature, as highlighted in the qualitative part of the study. In the qualitative study, our participants reported getting significant benefits even from short nature breaks, such as a few minutes in the garden. The qualitative results also indicated preferences in how highly sensitive individuals engage with nature: The majority expressed an overall preference for blue spaces, particularly the sea. A strong affinity with green areas and woodlands was also reported, and these findings were similar to previous findings by Black and Kern (2020).

However, a chaotic home environment did not affect flourishing more in highly sensitive individuals in the quantitative part of the study. Notably, chaos levels were generally low in this cohort. Nonetheless, the qualitative result corroborated the idea that a chaotic home environment decreases well-being in highly sensitive people. This discrepancy could be due to chaos affecting all individuals, including those with lower sensitivity, as shown by the main effect of CHAOS. It could also be due to the types of questions asked in the flourishing scale that are related to overall well-being in relation to one’s life. It is possible that a scale capturing stress would have provided a better quantitative tool to capture the relationship between high sensitivity and a chaotic environment. Alternatively, it is possible that, if other positive supports are in place, e.g., social support (Bonanno, 2004), or coping strategies, those who are highly sensitive could experience chaos as a growth factor. Fredrickson’s (2004) Broaden-and-Build Theory of Positive Emotions suggests that experiencing a range of emotions, including those elicited by chaotic circumstances, can broaden one’s repertoire of thoughts and actions, contributing to greater personal growth and resilience. The ability of our interviewed participants to cope better with their sensitivity and to adapt to their environment or accept their own reactions could potentially align with this view. Further research could test these different hypotheses.

Finally, the exploratory interaction between HSP-12, age, and nature connectedness indicates that nature connectedness plays a more important role in flourishing for highly sensitive individuals in middle and older age compared to those with lower sensitivity. In contrast, for the younger group (40–49), nature connectedness is a significant predictor of flourishing, regardless of the level of sensory processing sensitivity. This aligns with research suggesting that well-being trajectories change with age, often increasing in later life due to improved emotional regulation and a greater focus on meaning-making (Carstensen et al., 1999; Scheibe and Carstensen, 2010). HSP-12 significantly negatively predicted flourishing in all age groups; however, nature was a moderator only in the 50–59 and 60+. This may indicate that some highly sensitive people can avail of their connectedness with the natural environment to increase their flourishing; however, this is not the case for all. In addition, recent research has shown nature connectedness to be a mediating factor in the relationship between environmental sensitivity and mental health (Mariani Wigley et al., 2025). Studies have shown that nature connectedness plays a significant role in well-being and may become more meaningful with age, possibly due to a greater appreciation for natural beauty and an increased sense of belonging in nature (Lumber et al., 2017). The interviews support the idea that nature is perceived as a fundamental part of life, and given the age of participants, this may also suggest that with life experience, highly sensitive people adopt ways of being that are more compatible with their sensitivity. This is consistent with literature indicating that sensory processing sensitivity interacts with environmental influences on well-being, particularly in later life (Greven et al., 2019). Cultivating a relationship with nature may be one such adaptive strategy that contributes to flourishing in highly sensitive individuals as they age.

A main strength of this study was the combination of quantitative and qualitative methods with the quantitative results showing that increased nature connectedness predicts increased flourishing in SPS and the qualitative results elucidating what type of nature works best and what the benefits are. This study also helps to address the imbalance of previous research on the more negative aspects of SPS and, in the qualitative interviews, gives voice to a group that may not be heard as often as others. Some limitations of the study include the fact that the participants are mostly from white, educated, industrialised, rich, democratic cultural backgrounds, and it would be valuable to replicate the study with a more culturally diverse cohort. In addition, this study did not collect information on participants’ backgrounds or childhoods, which would add to the findings as childhood experiences in SPS have been found to be linked to psychological outcomes for adults with SPS (Aron et al., 2005; Booth et al., 2015).

While our findings highlight the importance of nature connectedness for flourishing in highly sensitive individuals, it is likely that the extent of these benefits may depend on factors such as the amount of time spent in nature (Mariani Wigley et al., 2025) and accessibility to natural spaces. Individuals with greater access to nature or those who actively spend more time in natural environments may experience stronger benefits compared to those with limited exposure. Future research could explore the role of these factors, examining whether differences in frequency and type of nature exposure (see Holzer et al., 2024) further moderate the relationship between sensitivity and flourishing. In addition, our qualitative sample consisted of individuals who met specific inclusion criteria relevant to our broader research questions. As a result, the findings may reflect perspectives shaped by these characteristics. Future research could benefit from including a more diverse sample in terms of demographic background, socioeconomic status, and geographical location to better understand how different groups experience the relationship between sensitivity, nature connectedness, and flourishing.

As positive psychology is the study of ‘what works’ in support of psychological well-being and flourishing (Seligman, 2011), further research on those people who are both highly sensitive and flourishing would help to increase understanding of how well-being can be achieved and maintained in SPS. Nature connectedness appears to be a potential psychological factor.

## Data Availability

The data presented in the study are included in the article/Supplementary material, further inquiries can be directed to the corresponding author.
