# Supplementary material for: Flourishing as a highly sensitive person: a mixed method study on the role of nature connectedness and chaotic home environment
Source: Front Psychol. 2025 Apr 16;16:1480669. doi: 10.3389/fpsyg.2025.1480669 (PMC12040826; doi:10.3389/fpsyg.2025.1480669)
Supplement: Supplementary file 1 [file Supplementary_file_1.docx]

Supplementary Material

# Interview Questions

Prior to knowing that you had high sensitivity, how did you view yourself in terms of being sensitive as compared to others?

Can you tell me about times within your home environment when you find things difficult? How do you feel in this situation? Are these feelings intense?

How do you help yourself when things get difficult?

Can you tell me about times within your home when you feel relaxed and calm in yourself?

How often do you spend time in nature? What are your favourite places in nature? How do you feel when you are in Nature? What benefits do you get?

How do you feel after you have been in nature?

What difference does it make to you to know that being highly sensitive is an biologically based innate personality trait?

Moving forward what difference will it make to you to know this?

# Thematic Analysis and coding

## Theme 1: Being sensitive can be challenging

| Emotional Reactivity and Physical Expression | **“**When younger would have described self as | P1 |
| --- | --- | --- |
|  | sensitive or odd compared to others” |  |
|  |  |  |
|  | “Felt separate from others” | P2 |
|  | “Always had a sense of not fitting in and wondering | P7 |
|  | why others weren’t like me” |  |
|  | “I would be more excited about things than other | P10 |
|  | people or brood on things more than other people” |  |
|  |  | P12 |
|  | “I noticed things other people didn’t” |  |
|  | “would have taken things the wrong way if something | P1 |
|  | was said or something happened” |  |
|  | “would feel more emotional about things than others” | P2 |
|  | “would feel emotional about things that happened in | P2 |
|  | real life or in movies” |  |
|  | “I do take things to heart” | P3 |
|  | “her bladder is very close to her eyes” (said about her) | P4 |
|  | “The first time I had to read at mass, I fainted” “that’ll |  |
|  | tell you the level of sensitivity” |  |
|  | “get annoyed by what others consider innocuous | P5 |
|  | noises”“can’t bear it, can’t bear it” |  |
|  | “if was given out to [as a child] would have taken i to | P6 |
|  | heart” |  |
|  | “cry in an instant” | P7 |
|  | “I’m incredibly emotionally sensitive” |  |
|  |  | P9 |
|  | “arguments affect my mental balance” |  |
|  | “feel a sense of danger and need to move away from |  |
|  | it”(arguments) |  |
|  |  | P10 |
|  | “When I was younger, my mood seemed to be up and |  |
|  | down” “I would find things really, really exciting or |  |
|  | really really devastating” |  |
|  | “I feel things so deeply” |  |
|  |  | P11 |
|  | “would have been more sensitive to the cross word” |  |
|  |  | P12 |
|  | “If the teacher made a joke at my expense would just |  |
|  | want the whole world to disappear” “they didn’t have |  |
|  | to say much for it to happen” |  |
| Thought rumination | “would ruminate in head over things for a long time” | P1 |
|  |  |  |
|  | “Sometimes I don’t like walking in silence” “my | P3 |
|  | head, everything starts going around” |  |
|  | “I take things to heart, as a young teacher, if there was | P4 |
|  | an issue, I took it to bed with me, it was in my |  |
|  | dreams, it wrecked me” |  |
|  |  | P6 |
|  | “rather than bouncing back would think things |  |
|  | through” |  |
|  |  | P8 |
|  | “I would have internalised emotions a lot“I have a |  |
|  | tendency to ruminate over injustice” |  |
|  |  | P10 |
|  | “I’d brood on things more than other people” |  |

## Theme 2: Stressful home environment

| Overwhelmed by demands | “ Being scheduled is stressful, 100%” | **P1** |
| --- | --- | --- |
|  | pottering, just pottering with no agenda” “It’s |  |
|  | stress free”“being on my own, I like being in my |  |
|  | own company” |  |
|  | “Sometimes, I feel overwhelmed, I’m just give, | P3 |
|  | give, give with nothing in return” |  |
|  | “too long a list to do” “feels overwhelming” | P2 |
|  | “When I’m calmest the schedule’s done” | P5 |
|  | “I’m not the kind of person that could run on busy, | P11 |
|  | busy, busy all the time, I can do it for a day or |  |
|  | two” “then there has to be a quiet day just to |  |
|  | backpedal a bit” |  |
|  |  | P7 |
|  | “I don’t like having to rush from one thing to |  |
|  | another, having no time to stop and gather myself” |  |
|  |  | P10 |
|  | “ I like to have breaks between each session, I |  |
|  | don’t like if I have to do loads and loads of things |  |
|  | all in one go” “I’d like to have to stop and gather |  |
|  | myself”, like to have gaps in between””find it |  |
|  | easier to pace myself a bit” |  |
| Overwhelmed by Noise | noise of phone is irritating” | P1 |
|  |  |  |
|  | “I need my space”, need a certain amount of peace | P2 |
|  | and quiet in my home” |  |
|  | “I cannot cope with too much noise, I cannot cope | P3 |
|  | with two noises together like if the TV is on and |  |
|  | the radio” |  |

|  | “my sister says, mine is the house where we have |  |
| --- | --- | --- |
|  | to suck crisps” “As for someone eating an apple, |  |
|  | Oh my god, that’s a whole other level” |  |
|  |  | P5 |
|  | “noise is definitely a trigger for me” “honestly, |  |
|  | breathing, chewing, paper rustling” |  |
|  | “I can’t bear it (noise of eating), Nan eating mint |  |
|  | imperials “can’t bear it, “can’t bear it” |  |
|  |  | P7 |
|  | “if someone is staying in my house and they put |  |
|  | the radio on in the morning” “I just don’t want to |  |
|  | hear it” Gets very annoyed and irritated |  |
|  | “If the radio is on and someone tries to talk to me, | P7 |
|  | I’m like hang on a minute, my brain is in two |  |
|  | different areas” |  |
|  | “if someone is listening to something on internet, |  |
|  | tv etc. late at night, it disturbs me” |  |
|  |  | P8 |
|  | “I prefer the house to be quiet, I would have radio |  |
|  | off, whereas if my wife was here she would have it |  |
|  | on” | P9 |
|  | “the television, the television, I could live without |  |
|  | a television perfectly happy, yeah the noise of it |  |
|  | would stress me out” | P10 |
|  | “Noise, sometimes it hurts my ears sounds, my |  |
|  | oversensitive days, everything is too bright, too |  |
|  | loud, it has a knock on effect on my mood””makes |  |
|  | me feel agitated” “that fight or flight feeling” |  |
|  |  | P3 |
| Discomfort with Conflict |  |  |
|  | “the kids aruging” “they were arguing and I wasn’t |  |
|  | getting anywhere with them, I was overwhelmed, I | P7 |
|  | just had to go” |  |
|  | “I can’t watch drama” (on TV) “like my adrenalin | P8 |
|  | is through the roof” “I find it hard to separate it” |  |
|  | “Don’t like raised voices” |  |
|  |  | P9 |
|  | “arguing, when I hear people that stresses me” “It |  |
|  | affects my mental balance and that’s the truth” |  |
|  |  | P11 |

|  | “any arguments, I would find very stressful” “I feel as if all my nerve endings are jangly for a while”“yeah I definitely don’t like raised voices” “People being out with each other can be uncomfortable for me” |  |
| --- | --- | --- |

## Theme 3: Enhanced Coping with Maturity

| Growth through mindfulness practices | “I’ve adapted in my own little ways, my own |  |
| --- | --- | --- |
|  | defensive systems maybe and adapters” |  |
|  |  | P1 |
|  | “I’m studying mindfulness” |  |
|  | “I’ve done coaching, so there’s elements that I |  |
|  | have picked up that helped me and support me” |  |
|  | “In mindfulness, there is a thing called |  |
|  | anchoring”, “If I can remember, 80% of the time, |  |
|  | maybe I can drop into my anchor where I feel |  |
|  | safe” |  |
|  | “I do a little bit of meditation and QiGong” |  |
|  | “I go to quiet places when I need solitude in |  |
|  | nature” |  |
|  | “ I went to counselling” (at a difficult time) | P4 |
|  | “I had to start doing meditation and deep |  |
|  | breathing” |  |
|  | “I brought meditation into my daily life so I |  |
|  | would be able to function” “It worked, it helped |  |
|  | me greatly” |  |
|  | “I do Yoga and that’s a quiet practice,” | P5 |
|  | “I work on a few things like that, you know, I | P6 |
|  | work on my contentedness”“I read books, self |  |
|  | awareness and not to label it, the self-help yes” “I |  |
|  | am interested in knowing all that” |  |
|  | “I love growing and I love learning and stuff like |  |
|  | that” |  |
|  | Have “self-awareness and awareness of the way I |  |
|  | am” “I would have an awareness of it, being |  |
|  | sensitive you know” |  |
|  |  | P7 |

|  | “I have been on what I’d call a spiritual journey |  |
| --- | --- | --- |
|  | for 15 years, so that’s when I would have been |  |
|  | conscious of what I was doing” |  |
|  | “I can do reiki and energy healing on myself” |  |
|  |  | P8 |
|  | “I meditate, I take maybe 10-15 minutes out and |  |
|  | that helps me” Came to meditation through “I |  |
|  | suppose it was an interest in mysticism, my |  |
|  | background is in engineering |  |
|  | “The calm and peace the mediative state reveals is |  |
|  | very valuable to me” |  |
|  |  | P9 |
|  | “I would be very much towards emotional |  |
|  | connection with people |  |
|  | I took up mindfulness, probably about 12 years |  |
|  | ago” |  |
|  | to be honest, “it was the best find ever” | P10 |
|  | “I found I was burning out, because I didn’t have |  |
|  | the balance right”, “I find it easier now just to sort |  |
|  | of pace myself a little bit” |  |
|  | “I meditate” |  |
|  |  | P1 |
| Self-acceptance | ” When was younger would have said |  |
|  | “very sensitive”, “And probably as I’ve gotten |  |
|  | older would probably say less so” “Maybe that’s |  |
|  | “more of an understanding of who I am” |  |
|  | “Age and life experience, “If I can handle the big | P2 |
|  | stuff, (loss of significant loved ones) why? Why |  |
|  | sweat the small stuff” “happy in my own |  |
|  | authentic little self” | P3 |
|  | “Well, I suppose, as I’ve gotten older, I’m better |  |
|  | with it, but probably when I was younger it was |  |
|  | harder” |  |
|  |  | P4 |
|  | “Life does toughen you up as you know, and life |  |
|  | experiences, you do get tougher like, but |  |
|  | underneath, you are still the same. |  |
|  | “I’ve trained myself as I’ve gone along to become |  |
|  | more confident about the things, that I am |  |
|  | sensitive to”“Now I know what the worst stress |  |
|  | can be” (Son’s medical emergency) “I measure |  |
|  | things against that”, “you know put it into |  |
|  | perspective” |  |

|  | “less emotionally sensitive, because I managed a | P5 |
| --- | --- | --- |
|  | big team of people” “And you develop a hardness |  |
|  | through work, you know,” cause you are being |  |
|  | paid to do a job and you have to do it” |  |
|  |  | P6 |
|  | “I’ve adapted in my own little ways, my own |  |
|  | defensive systems maybe and adapters” |  |
|  | Situation “where didn’t feel you should belong |  |
|  | there to a certain extent” “I’m not pushed about |  |
|  | that any more” |  |
|  | “ I think I’m learning a bit more to sort of stand | P7 |
|  | up and speak out” |  |
|  | “I used to work in the financial world, I knew my |  |
|  | soul was slowly dying.” |  |
|  | “ I just want to enjoy my job and enjoy a balance | P8 |
|  | in my life, and I’m happy with that” Being busier |  |
|  | and more ambitious at work “I know it wouldn’t |  |
|  | make me happy because I tried it and it didn’t” |  |
|  | “probably avoid a lot of them, (environments) that | P3 |
|  | I know will be hectic or busy” |  |
|  | “I can see three or four things down the road I | P4 |
|  | prepare for all eventualities and nearly I'm sorting |  |
|  | them as I'm going along. You know what I mean? |  |
|  | I can I see what's coming down the road 4 |  |
|  | try to pre-empt and avoid situations like that” | P6 |
|  | (stressful) |  |

## Theme 4: Nature as a vital requirement

| Nature is essential | When stressed “seek, solitude and nature”When | P1 |
| --- | --- | --- |
|  | husband was in the hospice “Every morning I would |  |
|  | go outside to the courtyard, there would be shrubs |  |
|  | and flowers” “If I didn’t get to go out there morning |  |
|  | and evening, I don’t know how I would have been |  |

|  | able to cope” “I’m not too sure what I would have |  |
| --- | --- | --- |
|  | done” |  |
|  | Especially on a busy day “I would need it, even if it’s |  |
|  | just sitting in the garden for 10/15 minutes” |  |
|  | “I hate the feeling of being hemmed in, I need nature | P2 |
|  | definitely, need access nature close to my home” |  |
|  | “Exercise, cycling, walking, walking definitely” |  |
|  | Greenery and listening to the birds, that kind of thing |  |
|  | helps” |  |
|  | “Being by the sea, definitely helps me”“I go walking, | P3 |
|  | I go out to get my headspace” |  |
|  | “fresh air, nature” | P4 |
|  | “I play tennis, you know it’s a great coping strategy, |  |
|  | belt that ball across the net, you forget everything, it |  |
|  | takes everything out” |  |
|  | “I go for a 6km walk every morning before |  |
|  | work”“walking and chatting with a friend |  |
|  | “Gardening”, I do need that downtime and it | P5 |
|  | completely fits” “I think its the peacefulness of it, I |  |
|  | guess that I like” |  |
|  | “Go to the sea” “going sailing in my little boat” | P6 |
|  | “I do things like go outside and stand barefoot on the |  |
|  | earth” “just let any tension or stress that I’m picking | P7 |
|  | up, go into the earth” |  |
|  | “I gotta stop and put my feet in the stream” (after |  |
|  | being in the city for a few days)” |  |
|  | Occasionally, I have had to just go for a walk |  |
|  | because, I’m just completely out of ideas”, so I just | P8 |
|  | need to stop and leave” |  |
|  | “pot plants in the backyard, comforting to look at” |  |
|  | “I walk, Iove to walk, I love to walk in nature” | P9 |
|  | Everything about it soothes my body and mind” |  |
|  | “If I am stressed when I come out” (of work), “then I |  |
|  | will drive out to Clonea” (beach). |  |
|  | “I feel much better when I’m outside. I always have | P10 |
|  | my windows open, even all through winter” |  |
|  | “A walk on the beach helps to maintain balance from | P11 |
|  | a stressful job” |  |

|  | “my escape plan (from a stressful situation) was just | P12 |
| --- | --- | --- |
|  | to go for a run everyday, along the beach |  |
| Nature's Rejuvenating Power | “110% something I enjoy” | P1 |
|  | Being in nature “grounds me” |  |
|  | “It’s like, you know, you plug in your phone to |  |
|  | charge. It’s a little bit like that for me, maybe, I just |  |
|  | feel depleted if I don’t have it |  |
|  | Spending 5 or 10 minutes in outdoor garden space |  |
|  | was better than counselling for me (when husband |  |
|  | was in hospice) |  |
|  | “I need time in nature, definitely” | P2 |
|  | “It makes me feel present, and I’m just kind of taking |  |
|  | in what’s around me and not worrying about other |  |
|  | things” “just being engaged with, the beauty, the |  |
|  | colour, the sounds”. |  |
|  | “Makes me feel reset” “nearly cleansed in a sense” |  |
|  | helps to create space in head |  |
|  | “It definitely, definitely helps me” (nature) “feel | P3 |
|  | calmer and more relaxed” |  |
|  | “Headspace wise, probably not often enough” |  |
|  | “I need to set myself up” “I’m getting better at it, I |  |
|  | think I’m kind of realising, I’m just kind of getting |  |
|  | burnt out at the moment” |  |
|  | Walking every morning “is the best therapy that I | P4 |
|  | have” “I walk 6k every morning before work” |  |
|  | “I’m rejuvenated coming home” (from the sea) |  |
|  | the physicalness of it is a thing, it is my go to thing” | P5 |
|  | (gardening) |  |
|  | “I need the downtime, so it completely fits” |  |
|  | (gardening) sense of accomplishment, sense of |  |
|  | achievement, it’s the peacefulness I like” |  |
|  | “I don’t need to climb Ben Nevis or anything like |  |
|  | that, I can get what I need out the back door” (in the |  |
|  | garden) “It’s naturally part of my routine” |  |
|  | Nature “is very very important, very important, I’ll | P6 |
|  | emphasise that ++, I just love nature |  |
|  | It brings “serenity” |  |
|  | “Nature for me is essential” “ I feel completely | P7 |
|  | spiritually connected to nature” “3 days without” (in |  |

|  | the city) “I was like, Oh my god, like you know, let |  |
| --- | --- | --- |
|  | me out, let me out” |  |
|  | It makes me feel amazing” |  |
|  | “it restores my equilibrium” |  |
|  | “I love to walk, I love to walk in nature” | P8 |
|  | “If I was disappointed or annoyed, going for a walk |  |
|  | by the sea and just listening to the crashing waves |  |
|  | was fantastic, very therapeutic” |  |
|  | It is a priorty”, (getting out to the beach for a walk) | P9 |
|  | “If I didn’t get outside for two or three days, I would |  |
|  | feel it in my mental wellbeing” |  |
|  | “I feel wonderful, it just calms everything you know” |  |
|  | “Everything just calms down, its that sense of space” |  |
|  | “most days you will find me at the beach for some |  |
|  | little periods of the day |  |
|  | I feel much better when I’m outside” “It changes | P10 |
|  | everything” (being outside in nature). |  |
|  | “I notice when Ive not been able to get out, I feel the |  |
|  | need to go somewhere |  |
|  | “If I don’t get out, I get more irritated by things, |  |
|  | things just annoy me more” | P10 |
|  | “It makes me sort of happier” |  |
|  | “I feel refreshed. I feel a lot more energised, |  |
|  | refreshed” “It’s almost like I’ve had a shower or I’ve |  |
|  | taken coats off” |  |
|  | “I notice the difference, if I don’t get out for a walk” | P11 |
|  | I’m more relaxed after it, more grounded”“Feeling of |  |
|  | connection with something bigger” “balances me” |  |
|  | I walk everyday, every morning” “35 minutes on the |  |
|  | beach before work” |  |
|  | If I didn’t have a garden, I would have to be near a |  |
|  | park or a river or a canal, somewhere I could just go | P12 |
|  | and get some nature” |  |
| Preferences for varying natural environments | “I’ve got a part-time job and the rest is the garden” |  |
|  |  |  |
|  |  |  |
|  | “The beach, the beach would probably be my go to |  |
|  | place” “ A quiet beach” | P1 |

|  | “I love the moon and the stars”Once I got my 10 |  |
| --- | --- | --- |
|  | minutes. |  |
|  | In the morning and in the night time. |  |
|  | Out in that little area (courtyard garden in a hospice) I |  |
|  | was, I was OK |  |
|  | “I would always have said the sea, being by the sea, |  |
|  | cause it’s that expanse, I’ve always been drawn to the sea” | P2 |
|  | “It depends on the feeling I get from a place” |  |
|  | “there are certain woodland areas that tick the box as |  |
|  | well” | P2 |
|  | “If I had to choose, I probably would be more drawn |  |
|  | to the sea” “there’s a peacefulness about it, I suppose |  |
|  | the feeling of (the sea) |  |
|  | Oh I’d love it, love it. I absolutely love sea water, I |  |
|  | love water. Love it. Yeah that would be my choice if | P3 |
|  | we were going anywhere overnight or for a weekend, |  |
|  | I’d always choose to be beside water” |  |
|  | I love the feel of the air, I love the sound of the water, |  |
|  | I love the sights everything I love. The smell, the sea, |  |
|  | I suppose all of your sense, just it, it justs hits the |  |
|  | right spot for me particulary water” |  |
|  | “ “there’s something very beautiful and there’s a |  |
|  | calmness about it when you are there at the sea” | P4 |
|  | “heartbeat starts to go with the tempo of the tide |  |
|  | coming in a out and you get that sense of calm” |  |
|  | “Gardening” It’s quiet, it’s productive, it’s |  |
|  | purposeful” “That would be my go to thing” | P5 |
|  | “I love hill walking, I love going out, you know in the |  |
|  | garden. I love the sea, the sea is my blue space.” “The | P6 |
|  | sea is my area, I love that” “I just love the sea” and “I |  |
|  | just love nature”. |  |
|  | “I love the way the sea can be different, I used to kind |  |
|  | of make the thing of it can be like you emotions, it |  |
|  | can calm one stage and it can be angry” “it’s just the |  |
|  | natural beauty and the noise as well” “Its just the |  |
|  | whole thing” |  |
|  | “I do love hillwalking, going to the mountains, I love |  |
|  | the forest, but I’d probably go for the sea first” |  |
|  |  | P7 |

|  | “I love to swim, I sea swim or lake swim. I didn’t |  |
| --- | --- | --- |
|  | think I could cope in the winter, but I managed to |  |
|  | change my mindset” |  |
|  | “I also love the Beech forests, in the spring and the |  |
|  | autumn” |  |
|  | “If I need to absolutely release, I just get into the |  |
|  | water” | P8 |
|  | “If I need grounding or to make me smile, then it’s |  |
|  | the forests and the green” |  |
|  |  | P9 |
|  | “If I get stressed, I’m drawn to the sea” |  |
|  | “everything about it” the beach “the sound of it, the |  |
|  | smell of it, the sight of it. Everything about it soothes |  |
|  | my body and mind“ I don’t have a garden” “but I |  |
|  | have lovely pots with lovely colour, you can sit at the |  |
|  | kitchen table eating your meal and look out” “It’s |  |
|  | peaceful and it’s nice” |  |
|  | “I love the woods, I love the mountains, but the sea |  |
|  | would be my first one” Its that motion of breathing, |  |
|  | its coming in and going out, and it’s so gentle, it |  |
|  | never stops, it just comes and goes” |  |
|  |  | P10 |
|  | I’m not far from a big park and I like going up there. |  |
|  | I like being by the water as well” |  |
|  | Woods, I like going around in the woods, that would |  |
|  | be my favourite” “I think it’s because it’s sort of cool |  |
|  | and it’s damp and it smells nice, there’s lots of |  |
|  | interest” |  |
|  |  | P11 |
|  | “Ooh I’d like the sea, I’ve grown up beside it, I like |  |
|  | the look, the sound of it and I like swimming, so that |  |
|  | would probably be my preference” |  |
|  | “But any nature really, I love trees, woodlands” |  |
|  | “There is something very replenishing, when you are |  |
|  | walking through that kind of area” |  |
|  |  | P12 |
|  | “Hills and we can, you know, 10 minutes walk to get |  |
|  | onto a foot path up the hills. So we are really, really |  |
|  | lucky and we do that a lot. “ |  |
|  | “I’ve got a part-time job and the rest is the garden” |  |

## Theme 5: It helps to understand SPS as an innate personality trait

| **If only; regrets about not** | “Over ten years ago, it would have made a vast difference, “If I had known as a young adult, it would | P1 |
| --- | --- | --- |

| **knowing about** | have made a huge difference, Huge” “If I had known, I |  |
| --- | --- | --- |
| **SPS at a younger** | wouldn’t have been comparing myself to others, that is |  |
| **age** | not as important to me now but it was back then” |  |
|  | “It would have made a difference to have known sooner | P2 |
|  | for life choices. Would have chosen a different job”. |  |
|  | “If I had seen it as a strength and followed that I would |  |
|  | have made better choices”. |  |
|  | “Would have found it easier to leave places didn’t want |  |
|  | to be or not to go at all”. |  |
|  | “I think it would have been useful to know when I was | P5 |
|  | younger, so instead of dealing with stuff to the detriment |  |
|  | of my own comfort , I probably would have spoken a bit |  |
|  | more about it” | P7 |
|  | “it probably would have been helpful to understand |  |
|  | myself a bit better and for life not just to be such a | P12 |
|  | struggle, to be more comfortable with myself.! |  |
|  | “It makes me wish I had trusted myself more when I was | P1 |
|  | younger” |  |
|  | “to recognise what I am like, so it is a little bit of |  |
|  | affirmation for me” |  |
|  | “That it is not something in me, it is a recognised trait, |  |
| **It’s a** | definitely helps to know that” |  |
| **thing thing! Self-** | “Before the questionnaire and this interview, I would | P2 |
| **acceptance and** | have been asking myself, what is wrong with me” |  |
| **understanding** | “Being able to know it and accept my true self” |  |
|  | “Knowing the numbers indicates there are a lot of |  |
|  | people just putting on a face”, |  |
|  | “While it helps to know there are others feeling the |  |
|  | same, it also helps to know that the other 70% dont and |  |
|  | when people say they understand how you feel, they |  |
|  | often acutally dont, it kind of helps to know that, like | P3 |
|  | knowing why they don’t understand acutally helps.” |  |
|  | “Knowing about it makes me feel like I dont have to |  |
|  | deny my needs. Beating self up less, will work more on |  |
|  | self-compassion around it” |  |
|  | “It makes me feel better for the simple reason that, oh it | P3 |
|  | is real its a thing, because when you do have people |  |
|  | saying things to you like, you’re oversensitive, and they |  |
|  | dont say it in a nice way, they say it as a criticism” |  |
|  | “Its good to know that its a thing and that there are other |  |
|  | people there with that” |  |

|  | “It will help me put things more into perspective, |  |
| --- | --- | --- |
|  | sometimes you feel crazy because of your thoughts, but |  |
|  | knowing its a real thing really helps”. | P5 |
|  | “I dont feel as odd about it, its not just me, its not a (says |  |
|  | name) thing, its a thing thing”, | P6 |
|  | “Knowing it is good, it is beneficial, it is ok and happy |  |
|  | that its 30% it helps with acceptance of who I am.” | P7 |
|  | “It s nice to think I am not strange, that its a biological |  |
|  | thing, that it is not just me being weird” | P9 |
|  | “I think it is lovely to be highly sensitive, I was |  |
|  | wondering if it means I am odd or contrary, but now I | P10 |
|  | see it as a positive trait, I like being highly sensitive!!” |  |
|  |  | P11 |
|  | “Makes me feel more relieved because you think there is |  |
|  | something wrong with you **“** |  |
|  | “For me having a new found awarenes about it will | P12 |
|  | certainly help me to stop comparing myself negatively |  |
|  | to others who are more outgoing and talkative” |  |
|  | “Can identifiy in her daughter, who suffers from |  |
|  | anxiety, incredibly talented and really good at |  |
|  | everything she does, but yes, she doesn’t rate herself at |  |
|  | all. It would be helpful to have more information to talk |  |
|  | to her about it” |  |
| **On wards and** |  |  |
| **upwards; feeling** |  | P1 |
| **more empowered** |  |  |
| **and confident** | “Instead of judging it in myself, I will embrace the |  |
| **about the future** | uniqueness” |  |
|  | “Will read and learn more about it, particularly coping |  |
|  | mechanisms around it” | P2 |
|  | “knowing now that it is a positive trait that changes |  |
|  | things, puts a more positive spin on it” “ would want to |  |
|  | explore it more.” |  |
|  | “Didnt really trust my intuition before but will more |  |
|  | now”, | P3 |
|  | “It will definitely make me feel better about myself, |  |
|  | understand myself more”. |  |
|  |  | P5 |

|  | “being able to explain to others that you are not just |  |
| --- | --- | --- |
|  | being annoying, is very helpful I think”.” It has already | P6 |
|  | made a difference (since doing the questionnaire and |  |
|  | reading up about it) being able to communicate it” |  |
|  | “I think I will change knowing, will be a little bit more |  |
|  | happy that I have just found that out. I think it will | P7 |
|  | improve things.” |  |
|  | “My ex boyfriend, always said I was too sensitive and |  |
|  | too this and too that and now I can have great pleasure |  |
|  | in telling him, this is how I am, this is how I’m made, so | P7 |
|  | its making me smile in a way” |  |
|  | “Yes I will be more empowered now to say it when I |  |
|  | need to , that its part of my make up that I cant cope |  |
|  | with those sort of things”. | P9 |
|  | “Absoutely empowering to know about it” |  |
|  | “I think it is like having an other string to my bow |  |
|  | knowing that I have this trait. It is empowering, I know |  |
|  | now it is an inner strength, a skill that I have, that helps | P10 |
|  | me to stay stable and to support other people”. |  |
|  | “Knowing will help me appreciate it more, rather than |  |
|  | thinking of it as a weird thing, its how my brain works |  |
|  | or processes things, now its a case of thinking oh yeah |  |
|  | that is how my brain works and appreciating it more and |  |
|  | appreciating that I do appreciate things in life and find |  |
|  | more joy and worry less if others dont feel the same” |  |

# Supplementary Figures and Tables

Supplemental Table 1

*Regression Model: HSP Group, NCI, HSP*NCI Interaction for age group 40-49*

| Predictor | B | SE B | b | t | 95% CI | | p |
| --- | --- | --- | --- | --- | --- | --- | --- |
|  |  |  |  |  | LL | UL |  |
| (Constant) | .68 | .16 |  | 4.42 | .38 | .99 | <.001 |
| HSP Group | -.42 | .07 | -.28 | -5.72 | -.56 | -.27 | <.001 |
| NCI | .33 | .05 | .32 | 6.65 | .23 | .43 | <.001 |
| HSP*NCI | .05 | .05 | .05 | 1.03 | -.05 | .14 | .306 |

Supplemental Table 2

*Regression Model: HSP Group, NCI, HSP*NCI Interaction for age group 50-59*

| Predictor | B | SE B | b | t | 95% CI | | p |
| --- | --- | --- | --- | --- | --- | --- | --- |
|  |  |  |  |  | LL | UL |  |
| (Constant) | .40 | .16 |  | 2.58 | .10 | .71 | .010 |
| HSP Group | -.23 | .08 | -.17 | -3.06 | -.38 | -.08 | .002 |
| NCI | .34 | .06 | .33 | 6.06 | .23 | .45 | <.001 |
| HSP*NCI | .17 | .05 | .19 | 3.62 | .08 | .27 | <.001 |

Supplemental Table 3

*Regression Model: HSP Group, NCI, HSP*NCI Interaction for age group 60+*

| Predictor | B | SE B | b | t | 95% CI | | p |
| --- | --- | --- | --- | --- | --- | --- | --- |
|  |  |  |  |  | LL | UL |  |
| (Constant) | .66 | .19 |  | 3.46 | .28 | 1.04 | <.001 |
| HSP Group | -.18 | .09 | -.17 | -2.00 | -.36 | -.00 | .047 |
| NCI | .26 | .07 | .33 | 3.90 | .13 | .39 | <.001 |
| HSP*NCI | .16 | .07 | .19 | 2.23 | .02 | .31 | .028 |
